# Is Perceived Athlete Leadership Quality Related to Inside Sacrifice and Perceived Performance in Team Sports? The Mediating Role of Team Identification

**DOI:** 10.3389/fpsyg.2021.662250

**Published:** 2021-06-21

**Authors:** Miguel A. López-Gajardo, Juan J. Pulido, Miguel A. Tapia-Serrano, Iván Ramírez-Bravo, Francisco M. Leo

**Affiliations:** ^1^Department of Didactics of Musical, Plastic and Corporal Expression, Faculty of Sport Science, University of Extremadura, Cáceres, Spain; ^2^Department of Didactics of Musical, Plastic and Corporal Expression, Faculty of Teacher Training, University of Extremadura, Cáceres, Spain

**Keywords:** group dynamics, athlete leadership, social identity theory, sport psychology, team sports

## Abstract

The study aimed to analyze the relationship between athletes' perceptions of athlete leadership quality, team identification, inside sacrifice, and performance. A total of 299 players of collective sports (soccer, beach soccer, basketball, volleyball; *M*_age_ 19.05, *SD* = 5.10) participated through a cross-sectional design survey. Data were analyzed using structural equation modeling. Results highlight the positive relationships between perceived quality of athlete leaders, inside sacrifice, and perceived performance, and between inside sacrifice and perceived performance. Furthermore, inside sacrifice perceived by the athletes was a positive mediator between perceived athlete leadership quality and perceived performance. Also, team identification was a positive mediator in the association between inside sacrifice and perceived performance. These findings extend knowledge about the athlete leadership quality context. These results can also be useful for further research and implications in team sports' performance, as coaches and sports psychologists would have more information about their teams' perceptions of leadership quality to achieve positive outcomes in players' inside sacrifice and performance. The findings also highlight the importance of developing team identification to improve the relationships between perceived athlete leadership quality, inside sacrifice, and perceived performance.

## Introduction

Coaches, players, and sports psychologists recognize the importance of leading athletes within a team. For example, Mourinho (2019), one of the best soccer coaches in the last few years said: “when you have them (i.e., team leaders), your team is one step ahead.” This quote by Jose Mourinho points out the importance for some coaches of building a good workgroup to promote good athlete leadership in the team. Good athlete leadership can turn the team into an effective operational group in terms of organization, teamwork, and performance (Fransen et al., 2015a).

### Athlete Leadership

Athlete leadership has been defined as “an athlete, occupying a formal or informal role within a team, who influences a group of team members to achieve a common goal” (Loughead et al., 2006, p. 144). Athlete leadership has usually been associated with formal team leaders, such as captains. However, according to Fransen et al. (2014), captains are not always the most influential players in the team. Whether the leader is formal or informal may not be so relevant; instead, the quality of that leadership can benefit the team (Cotterill and Fransen, 2016). The quality of athletes' leadership has been defined as that player leaders who fulfills well his or her specific role, who develop an impact on team functioning and are social well-accepted by teammates (Fransen et al., 2014). In fact, such leadership quality has been related to the leader's effectiveness (Fransen et al., 2017).

According to Loughead et al. (2006) and Fransen et al. (2014), there are different roles in athlete leadership. On the one hand, some leaders, called task and motivational leaders, are characterized by developing their leadership in training sessions and competition actions. The task leader helps the team to focus on the field, making tactical decisions and giving advice, whereas the motivational leader encourages the teammates' engagement in any situation on the field. On the other hand, other leaders, known as social and external leaders, are characterized by exercising leadership in an off-sports context. The social leader develops good relations within the team, creating a good atmosphere off the field, and the external leader acts as a link between the players and the club management, social networks, or sponsors. Therefore, the perceived athlete leadership quality of each of these leadership roles (i.e., task, social, motivational, and external) can improve teams' collective functioning (Price and Weiss, 2011, 2013; Fransen et al., 2017). Previous studies has shown that the optimal fulfillment of all of these four types of leadership together has demonstrated several benefits, such as team cohesion (Fransen et al., 2016a; Loughead et al., 2016), collective efficacy (Fransen et al., 2014, 2016a), or team confidence (Fransen et al., 2016a). Thus, we will focus on the general leadership quality grouping the best athlete leader in each of the four leadership roles. More comprehensive definitions of the four leadership roles (task, motivational, social, and external leader) can be found in Table 1.

**Table 1:** The definitions of the four leadership roles, as described by Fransen et al. (2014).

**Leadership role**	**Definition**
1. Task leader	A task leader is in charge on the field; this person helps the team to focus on our goals and helps in tactical decision-making. Furthermore, the task leader gives his/her teammates tactical advice during the game and adjusts them if necessary.
2. Motivational leader	The motivational leader is the biggest motivator on the field; this person encourage his/her teammates to go to any extreme; this leader also puts fresh heart into players who are discouraged. In short, this leader steers all the emotions on the field in the right direction in order to perform optimally as a team.
3. Social leader	The social leader has a leading role besides the field; this person promotes good relations within the team and cares for a good team atmosphere, e.g., in the dressing room, in the cafeteria or on social team activities. Furthermore, this leader helps to deal with conflicts between teammates besides the field. He/She is a good listener and is trusted by his/her teammates.
4. External leader	The external leader is the link between our team and the people outside; this leader is the representative of our team toward the club management. If communication is needed with media or sponsors, this person will take the lead. This leader will also communicate the guidelines of the club management to the team regarding club activities for sponsoring.

Research has shown that high-quality leaders are related to team members' efforts (Greenlees et al., 1999). When team leaders unite the team through their leadership methods and the team members sacrifice themselves for the team, the teams will achieve their goals more easily (Bandura et al., 2019). Prapavessis and Carron (1997) described sacrifice as “group members voluntarily initiating an action or giving up prerogatives or privileges for the sake of another person or persons” (p. 231). This variable is considered a voluntary behavior that encompasses several concepts such as empathy, altruism, cooperation, or loyalty (Prapavessis and Carron, 1997). These authors pointed out that the specific sacrifice within the context of practicing and competing is called inside sacrifice (i.e., players' sacrifice during daily practice and competition; Cronin et al., 2015). They also proposed that inside sacrifice involves both personal (e.g., sacrifices I make) and teammates' sacrifice (e.g., sacrifices my teammates make). Considering that teams constitute a collective context, where players are nested in sports teams, it is necessary to examine the personal sacrifice and behaviors they expect and perceive from team members (Cronin et al., 2015).

Decades of research have shown that sacrifice is associated with group processes (Zander, 1982; Cronin et al., 2015). Athletes' sacrifice has been strongly associated with coach leadership, coach-athlete relationships (Jowett and Timson-Katchis, 2005), and athlete leadership (Cronin et al., 2015). In this wave of research, inside sacrifice represents effort and the effect of good athlete leadership. If players perceive that their leader supports them, coordinates everyone's actions, and helps everyone to perform well, they will be more likely to put out effort for the team. Therefore, when leaders convince and persuade their teammates (Cotterill and Fransen, 2016), they may increase their inside sacrifice within the team (Bandura et al., 2019).

In this regard, although research analyzing athlete leadership in competitive sports has advanced, much work remains to be done. For instance, previous scientific evidence has shown the benefits of athlete leadership and determined which mechanisms may help to improve teams' positive outcomes (Fransen et al., 2014, 2016a, 2017, 2018; Loughead et al., 2016). However, we do not know whether perceived athlete leadership quality can encourage athletes to sacrifice themselves for the team. Hence, it would be interesting to examine the relationship between athlete leadership quality and players' reported inside sacrifice. Drawing on these previous investigations, we expect that the perceived quality of the athlete leaders within the team (i.e., task, motivational, social, and external leader) will be positively associated with players' inside sacrifice (Hypothesis 1).

Perceived athlete leadership quality could lead to other positive outcomes (Fransen et al., 2014, 2015a). Specifically, perceived athlete leadership quality has been associated with higher satisfaction with team performance (Crozier et al., 2013; Fransen et al., 2020b) or team effectiveness (Fransen et al., 2017). Thus, when team leaders perform their functions well, remembering which tasks must be performed, supporting their players on the field, promoting positive group relationships, and managing external aspects, the team will be more likely to perform well. Promoting athlete leadership is important to improve individual and team performance in team sports (Price and Weiss, 2011, 2013; Cotterill, 2013; Cotterill and Fransen, 2016; Fransen et al., 2017; Leo et al., 2019). Specifically, previous research found that the fulfillment of these four high-quality athlete leadership roles (i.e., task, motivational, social, and external) led to players' perception of better performance (Fransen et al., 2017) and a higher number of free throws scored by every player, or to less time needed to complete a task in an experimental study (Fransen et al., 2015a, 2016b, 2018). Thus, we expect that high-quality perceived athlete leadership will be positively related to players' perceived performance (Hypothesis 2).

Considering inside sacrifice and performance as benefits of perceived athlete leadership quality, we expect that these two variables will not be on the same level. When all team players sacrifice and strive during training sessions and matches, performance is expected to improve and team goals to be achieved (Boyd et al., 2014). Although several studies defend that individual sacrifice and group processes are related to team performance (Prapavessis and Carron, 1997; Phillips et al., 2010; Cronin et al., 2015), to our knowledge, there is no empirical investigation focused on inside sacrifice within a team and its relationship with performance in the sports context. Thus, our next aims refer to the relationship between inside sacrifice and performance perceptions, and whether inside sacrifice mediates the relationship between perceived athlete leadership quality and perceived performance. Assuming that perceived athlete leadership quality is linked to inside sacrifice and perceived performance and that sacrifice can determine perceived performance, we expect that inside sacrifice will mediate this relationship, as it fulfills the mediation postulates (Hayes, 2009). Also, as prior scientific evidence showed, inside sacrifice was a mediator between coach leadership (i.e., transformational and authentic) and collective behavior in group tasks (i.e., cohesion; Cronin et al., 2015; Bandura et al., 2019). Thus, we will examine the association between inside sacrifice and perceived performance and the underlying mechanisms of the mediation between inside sacrifice, perceived athlete leadership quality, and perceived performance. Hence, we hypothesize that athletes' inside sacrifice will be positively related to perceived performance (Hypothesis 3), and will positively mediate the relationship between perceived athlete leadership quality and perceived performance (Hypothesis 4).

We also seek to explain the underlying mechanism through which high-quality perceived athlete leadership can affect players' reported inside sacrifice, and in turn, their perceived performance. As mentioned, recent research has shown the positive effects of high-quality athlete leaders in different group dynamics applied in team sports (Fransen et al., 2014, 2015a, 2016a,b), where, according to the Social Identity Theory (SIT), team identification has significantly improved these effects (Fransen et al., 2014, 2015a, 2016b). The recently proposed SIT approach related to athlete leadership focuses on team identification as the essential key to influence followers (Haslam et al., 2011). Thus, leadership is a group characteristic that directly influences team identification (Ruggieri and Abbate, 2013).

Team identification is a concept within the framework of SIT. SIT refers to “that part of an individual's self-concept which derives from his/her knowledge of his/her membership of a social group (or groups), together with the value and emotional significance attached to that membership” (Tajfel, 1981, p. 255). Specifically, SIT proposes that people can define themselves depending on the specific context either as unique individuals (i.e., in terms of “I”) or as group members (i.e., in terms of “us”). These characteristics of SIT make players feel a part of the same group and they know what the group stands for (Haslam et al., 2011; Steffens et al., 2014; Fransen et al., 2015b). It is precisely their sense of themselves as part of “us” that “makes group behavior possible” (Turner, 1982, p. 21). In other words, effective leaders “don't think ‘I'. They think ‘team”' (Drucker, 1992, p. 14). The variable team identification has been employed in recent studies to measure this feeling of “us” (Fransen et al., 2014, 2016a).

Previous research has shown that team identification is a potential mediator between perceived athlete leadership quality and several group processes (e.g., collective efficacy or cohesion; Fransen et al., 2014, 2015b, 2016a). It has also been shown that if team leaders promote a sense of “we” and team ownership, this helps the group focus on its goals and keep striving for the best results (Fransen et al., 2020a). This means that when athletes perceive high-quality leaders on their team, they tend to feel more strongly identified with the team. Leaders will make the whole team share a collective belief to achieve the same objectives, generating a team feeling among all the players. Therefore, it is important to identify with the team to be more predisposed toward individual and collective sacrifice to improve team performance (Cronin et al., 2015). Although these associations have not yet been demonstrated in a sports context, some anecdotal quotes have hinted at their potential. For example, one of the best coaches of NBA, Jackson (2014), illustrated the importance of “we” to the team: “Good teams end up being great teams when their members trust each other to give up the ‘me' for the ‘we'.” This quote highlights the feeling of being a part of the same group (i.e., team identification; Haslam et al., 2011), improving cooperation, helping, and making greater efforts (Reicher et al., 2018; Stevens et al., 2019). At the same time, due to team identification, group members should be more willing to sacrifice themselves for the team to achieve their shared goals (Reicher et al., 2018; Stevens et al., 2019). According to findings in previous studies, we propose that team identification will mediate the relationship between perceived athlete leadership quality and players' inside sacrifice (Hypothesis 5a), and conjointly with players' reported inside sacrifice (i.e., team identification and inside sacrifice), they will mediate the relationship between perceived athlete leadership quality and perceived performance (Hypothesis 5b).

Thus, the current study attempts to extend the existing scientific knowledge of athlete leadership in two ways. First, we analyzed the impact of perceived athlete leadership quality on two types of collective outcomes: inside sacrifice and performance reported by players. Second, the present paper goes beyond the mere relationship between perceived athlete leadership quality, inside sacrifice, and perceived performance, seeking to explain several indirect mechanisms, such as team identification and inside sacrifice, through which these relationships occur.

## Method

### Participants

A sample of 299 athletes correctly completed the questionnaires, a response ratio of 93.32%. Following the exclusion criteria of Leo et al. (2019), 17 questionnaires (5.38%) were removed from the original sample of 316, due to invalid responses (i.e., not fully completed, the same item was answered several times, or due to a clear response pattern). They corresponded to 17 teams (soccer = 260; beach soccer = 14; basketball = 6; volleyball = 19) and were aged between 14 to 42 years (*M* = 19.05, *SD* = 5.10). Of the participants, 272 were male (*M* = 18.82, *SD* = 5.12) and 27 were female (*M* = 21.11, *SD* = 4.43). This study employed convenience sampling methods.

### Instruments

All items included in these scales were presented in the players' language (i.e., Spanish). To translate and adapt the instrument in the Spanish sport context, the authors followed the strategies proposed by Muñiz et al. (2013). First, a professional translator with 15 years expertise in sport psychology translated the instrument from English to Spanish. Second, two members of the research team— university professors with PhDs in Sport Psychology and an advanced level of English— individually analyzed each item using the checklist for the quality of the translation/adaptation of items designed by Muñiz et al. (2013). Third, two new experts—university professors with PhDs in Sport Psychology and an advanced level of English—analyzed the content of each item according to its domain representation, relevance, and clarity. Fourth, a pilot test was conducted with 12 players (soccer = 6; beach soccer = 2; basketball = 2; volleyball = 2) who found no problems in the content of the items.

#### Perceived Quality of Athlete Leaders

We examined perceived athlete leadership quality following a previous study of Fransen et al. (2014), that used a one-item measure to assess the overall perceived leadership quality of each of the four leaders within the team (task, motivational, social, and external leader; see Table 1) concerning their specific role. First, to identify the leaders, players were presented with a description of each leadership role. Second, they indicated which teammates best matched the description of each of the four leadership roles. Third, the quality of the four leadership types was evaluated. When the players had selected the teammate or teammates they considered a certain type of leader (task, motivational, social, and external leader), they rated the following item “To what extent do you think that this leader fulfills his role as leader well?” on a 7-point Likert scale, ranging from 1 (*very poorly*) to 7 (*very well*). Thus, participants were asked to indicate the perceived quality of the motivational, social, and external leader, concerning their specific role and comprised in one factor. A higher score on these items indicated players' perceived better quality of the athlete leaders within the team. Hierarchical Confirmatory Factor Analyses (H-CFA) established that the perceived quality of each of the four different leadership roles contributed to an overall measure of perceived athlete leadership quality. To evaluate model fit, scores >0.90 were considered acceptable for incremental indexes such as CFI and TLI (Hu and Bentler, 1999), and values lower than 0.08 for the RMSEA and SRMR (Browne and Cudeck, 1993): χ^2^(2) = 6.376, *df* = 2, *p* = 0.04; CFI = 0.97, TLI = 0.92, RMSEA = 0.06, 95% CI (0.00, 0.11), SRMR = 0.03. Results showed acceptable standardized factor loadings for task (λ = 0.68), motivational (λ = 0.76), social (λ = 0.85), and external leader dimensions (λ = 0.72). Internal consistency values were also adequate (α = 0.84, ω = 0.85; Nunnally and Bernstein, 1994).

#### Team Identification

Following previous research, this variable was measured using a total of five items for athletes included in one factor (Doosje et al., 1995; Boen et al., 2007; De Backer et al., 2011). These items were: “Being a member of the team is very important for me,” “I am very proud to be a member of this team,” “I am very happy that I belong to this team,” “I feel very connected with this team,” and “I identify strongly with this team.” Participants assessed each item on a 5-point response scale ranging from 1 (*strongly disagree*) to 5 (*strongly agree*). The one-factor CFA indicated an adequate fit: χ^2^(2) = 4.009, *df* = 3, *p* = 0.26, CFI = 0.99, TLI = 0.98, RMSEA = 0.03, 95% CI (0.00, 0.12), SRMR = 0.03. Regarding the factor loadings of the global factor, adequate values were obtained in all cases (λ = 0.51–0.99). The internal consistency of this identification scale was also adequate (α = 0.87, ω = 0.86; Nunnally and Bernstein, 1994).

#### Inside Sacrifice

Athletes' perceptions of inside sacrifice were measured with the Group Sacrifice Scale (GSS), originally designed by Prapavessis and Carron (1997), with a total of 16 items (e.g., “I am willing to carry out responsibilities I don't like for the good of the team”). Specifically, we used the personal and teammate inside sacrifice dimensions created by Cronin et al. (2015) based on GSS. Following to Prapavessis and Carron (1997) conceptualization as a main dimension of sacrifice, we decided to collapse into a general dimension involving the personal and teammate sacrifice factors. Athletes responded to all items on a nine-point scale ranging from 1 (*strongly disagree*) to 9 (*strongly agree*). An H-CFA model fit the data adequately: χ^2^ = 153.175, *df* = 71, *p* = 0.000, CFI = 0.922, TLI = 0.904, RMSEA = 0.062, 95% CI (0.049, 0.076), SRMR = 0.077. Factor loading values were adequate for personal (λ = 0.40–0.75) and teammate sacrifice factors (λ = 0.58–0.89). Both dimensions had adequate levels of internal consistency (α = 0.89, ω = 0.89; Nunnally and Bernstein, 1994).

#### Perceived Performance

In team sports, where there is a high number of interactions occurring in competitions, performance is a multifactorial variable and very difficult to measure. As a consequence, there is no standardized and validated instrument to analyze performance in the sports context. On the one hand, several researchers have used objective measures such as league standings (Heuzé et al., 2006). Although this might be useful for some studies, it can be problematic insofar as it could ignore the team's initial expectations and objectives, the actual context of the team, or the confounding contextual factors that are generated during a season (e.g., accumulation of injuries). On the other hand, other researchers have used players' self-reported ratings to analyze performance in team sports (Fransen et al., 2015b, 2017; Davis et al., 2018; Leo et al., 2019). According to Tenenbaum and Gershgoren (2011), this is an ecological and reliable measure to assess this variable in team sports. Therefore, for our study, the subjective perceptions of the performance of the players of each team were evaluated using the one-item scale of Dithurbide et al. (2009). On the one hand, athletes were asked to rate their team performance over the season (e.g., “the team's performance during the season has been.”). On the other hand, the same item was also adapted to measure the individual performance perceived by each athlete (e.g., “your individual performance on the team during the season has been.”). Both items were analyzed for a general dimension called perceived performance and rated on a five-point Likert scale ranging from 1 (*poor*) to 5 (*excellent*).

### Procedure

First, the study received the University Bioethics Committee's approval (application number 239/2019), thus following the Helsinki Declaration (1964). Also, all athletes were treated according to the American Psychological Association (2019) regarding consent, confidentiality, and anonymity of responses. Accordingly, the data would be accessed only by the investigators of the work and would be processed exclusively for the field of research. Second, the first author contacted the clubs' managers via email to inform them about all the objectives and stages and to find out which teams were interested in the project. Specifically, clubs were recruited via personal contacts and were required to compete in national leagues in Spain, corresponding to the following team sports: soccer, beach soccer, basketball, and volleyball. In total, the first author contacted 25 teams of which 17 accepted to participate (participant rate = 68%). Third, after they had agreed to participate in the study, all the athletes were informed of the procedure to be followed. In this stage, the first author of this investigation handed out the letter of information and requested informed consent from all senior athletes to participate in the project. For athletes under 18 years old, consent to participate in the study was signed by the player and the parents.

A cross-sectional quantitative design was used. Data were collected at mid-season, before a training session, through a paper survey. In this way, the athletes had developed an adequate perception of the target variables and could express a critical point of view about the context of the team's coexistence. The athletes were requested to complete the questionnaires individually and without distractions or the presence of any person associated with the club environment. They were supervised by the research assistants. The athletes completed the questionnaires in ~10 min. No rewards were given to players for participation in this research.

### Data Analysis

All statistical analyses were carried out using Mplus version 7.3 (Muthén, L. K., and Muthén, 1998–2017). Firstly, as preliminary analyses, we ran a CFA on each scale to determine acceptable model fit. Secondly, descriptive statistics, intraclass correlations, bivariate correlations, and reliability analysis were conducted. Thirdly, in the main analyses, structural equation modeling (SEM) was used to test the relations between perceived athlete leadership quality, team identification, inside sacrifice, and perceived performance. Subsequently, we used SEM to test the hypothesized and alternative models. The robust maximum likelihood (MLR) estimator was used, as it is robust for non-normal observations and can handle random missing data (Yuan and Bentler, 2000). We also controlled for potential group-level effects due to the between-team variance (ICC = 0.05–0.37; Hox, 2010) through the correction of standard errors of the parameters, using the Mplus COMPLEX instruction (Muthén, L. K., and Muthén, 1998–2017). The small sample of teams led us to test a model targeting the individual level of analysis. Finally, indirect effects were tested using the bias-corrected bootstrap method [10,000 samples with 95% bias-corrected confidence intervals (CIs); MacKinnon et al., 2004].

## Results

### Descriptive Statistics

Table 2 displays the means, standard deviations, reliability analysis, and correlations of the variables included in the investigation. Overall, the correlation analysis revealed positive relationships between all the study variables (*r* = 0.25–0.40, *p* < 0.001).

**Table 2:** Means, standard deviations, bivariate correlations, and reliability analysis of the variables.

	**M**	**SD**	**α**	**ω**	**1**	**2**	**3**	**4**
1. Perceived quality of athlete leaders	6.07	0.86	0.84	0.85	–			
2. Team identification	4.78	0.46	0.87	0.86	0.25^***^	–		
3. Inside sacrifice	7.76	1.19	0.89	0.89	0.33^***^	0.40^***^	–	
4. Perceived performance	4.13	0.66	–	–	0.34^***^	0.36^***^	0.40^***^	–

*** *p < 0.001*.

### Main Analysis

**Figure 1:**
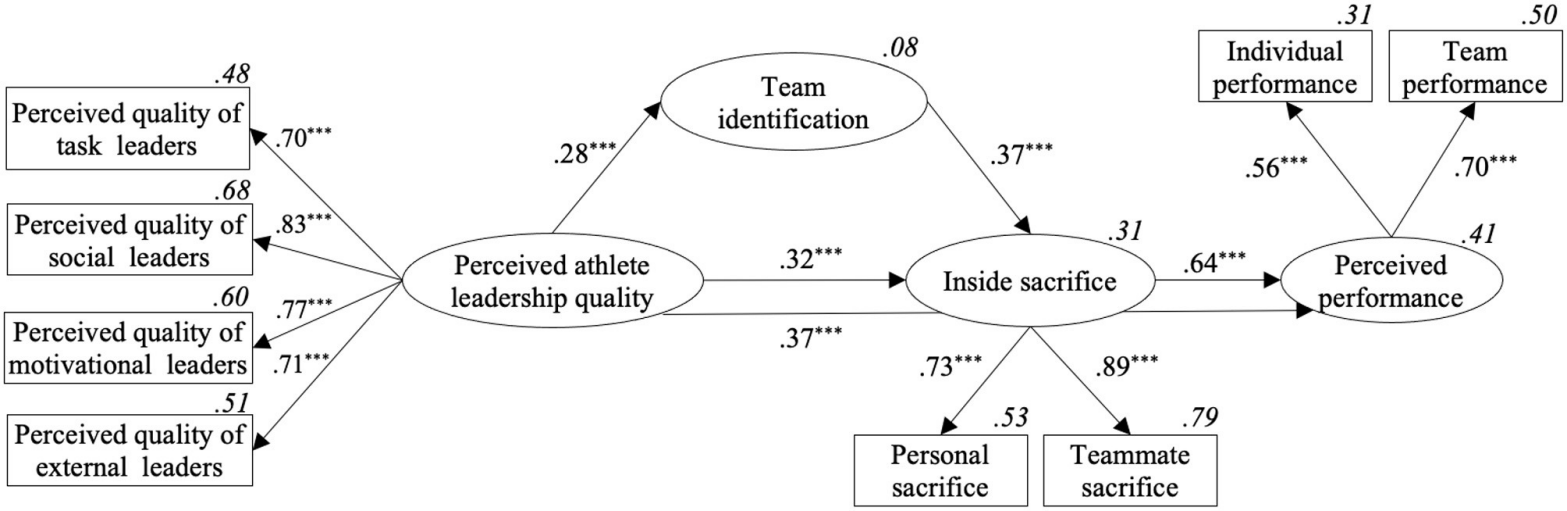
SEM of the relationships between perceived athlete leadership quality, team identification, inside sacrifice and perceived performance. ***p < 0.001. Proportions of explained variance are presented in italics.

SEM was used to test the different relationships among the variables represented in the model (see Figure 1). Specifically, perceived quality of athlete leaders was included as the independent variable, inside sacrifice as a mediator, and perceived performance as a dependent variable. Lastly, team identification was included as a mediator between perceived athlete leadership quality and inside sacrifice.

Firstly, the model showed an adequate fit to the data: χ^2^ = 43.391, *df* = 24, *p* = 0.009, CFI = 0.954, TLI = 0.930, RMSEA = 0.052 [95% CI (0.026, 0.076)], SRMR = 0.065. Secondly, standardized beta values showed that perceived athlete leadership quality was positively related to inside sacrifice (β = 0.32, *p* < 0.001) and perceived performance (β = 0.37, *p* < 0.001). Thirdly, inside sacrifice was positively associated with perceived performance (β = 0.64, *p* < 0.001) and had a positive and partial indirect effect on the relationship between perceived athlete leadership quality and perceived performance [β = 0.21, *p* < 0.001, 95% CI (0.10, 0.33)]. Finally, team identification presented a positive and partial mediation effect between perceived athlete leadership quality and inside sacrifice [β = 0.10, *p* = 0.002, 95 % CI (0.05, 0.18)], and, together with inside sacrifice (i.e., team identification and inside sacrifice), between perceived athlete leadership quality and perceived performance [β = 0.07, *p* = 0.011, 95% CI (0.02, 0.13)].

### Hypothesized Alternative Models

To ensure that the hypothesized model provided the best fit indices, two meaningful alternative models were tested (see Hershberger, 2006). First, in Model 1, we established team identification and inside sacrifice as two mediators at the same level. Accordingly, team identification and inside sacrifice were hypothesized as sharing covariance rather than representing a direct path between them. Second, in Model 2, we replaced the direct effect of perceived athlete leadership quality on sacrifice and established a model representing a linear process: (1) perceived athlete leadership quality, (2) team identification, (3) inside sacrifice, and (4) perceived performance. These models were tested because previous empirical evidence suggests that the main role of team identification (Fransen et al., 2014, 2015a, 2016b) and inside sacrifice (Bandura et al., 2019, Cronin et al., 2015) is mediation. However, to our knowledge, there are no previous studies that show how the two variables are associated, as they could operate jointly (Model 1) or at different levels (Model 2). Nonetheless, both alternative models showed a poor fit to the data {Model 1: χ^2^ = 61.503, *df* = 24, *p* < 0.001, CFI = 0.910, TLI = 0.865, RMSEA = 0.072 [95% CI (0.050, 0.095)], SRMR = 0.078; Model 2: χ^2^ = 59.600, df = 25, *p* < 0.001, CFI = 0.917, TLI = 0.881, RMSEA = 0.068, [95% CI (0.046, 0.090)], SRMR = 0.116}.

## Discussion

This study had five main objectives: (1) to analyze the association of players' perceived quality of athlete leaders with their perceived inside sacrifice, (2) to analyze the association between perceived athlete leadership quality and perceived performance, (3) to examine the relationship between reported inside sacrifice and perceived performance, (4) to explore the mediating effect of perceptions of inside sacrifice in the relationship between perceived athlete leadership quality and perceived performance, and (5) to test the mediating effect of perceived team identification in the relationship between perceived athlete leadership quality and inside sacrifice, as well as in the relationship between perceived athlete leadership quality and perceived performance, with team identification and reported inside sacrifice as mediators. Overall, we observed a positive relationship between perceived athlete leadership quality and inside sacrifice and a positive association between perceived athlete leadership quality and perceived performance. Furthermore, inside sacrifice was positively related to perceived performance and also acted as a positive mediator between perceived athlete leadership quality and perceived performance. Team identification also acted as a mediator between perceived athlete leadership quality and perceived performance. Finally, team identification and inside sacrifice acted as positive mediators between perceived athlete leadership quality and perceived performance. Thus, after analyzing the results obtained, these findings are conceptually consistent and robust, and are in line with previous research (Fransen et al., 2014, 2016b; Cronin et al., 2015), supporting all the hypotheses.

Firstly, regarding Hypothesis 1, the results showed that perceived athlete leadership quality had a positive association with inside sacrifice (Hypothesis 1). These results are in line with the findings of previous research (Ruggieri and Abbate, 2013), which found a significant relationship between effective leadership and workers' sacrifice in organizational contexts. However, this relationship had not been demonstrated in the sports setting. Hence, our findings provide further evidence of the benefit of an perceived athlete leadership quality approach in team sports settings. A possible explanation for this relationship is that leaders' inspirational motivation for their followers, who accept their leaders' collective view, is likely to promote these positive behaviors (e.g., commitment; Hodge et al., 2014; Fransen et al., 2017) and engage the teammates, correlating with high inside sacrifices (Cronin et al., 2015). Therefore, when athletes perceive their teammates as good leaders, they will probably sacrifice more to achieve team goals. In this regard, further research could consider examining leaders' behaviors that generate more inside sacrifice in team sports.

Secondly, concerning perceived athlete leadership quality and perceived performance (Hypothesis 2), a positive and significant association was found between the two variables (Slater and Barker, 2019). This positive relationship could due to leaders' ability to positively influence the group, encouraging them through actions, reminding them of the required tasks and the athletes' placement, or indicating when the team should apply pressure. The relationship of these interactions between the leader and the other athletes is the key to team performance (Crozier et al., 2013; Fransen et al., 2017). This influence has also been corroborated in experimental studies, showing that leadership extends throughout the team so that other team members are more self-confident and perform better (Fransen et al., 2015a, 2016b, 2018). Therefore, we conclude that high-quality team leadership can influence athletes (Fransen et al., 2016a) and promote optimal team effectiveness (Fransen et al., 2017), characterized by increased levels of inside sacrifice and perceived performance.

Thirdly, we found that inside sacrifice was positively associated with athletes' perceptions of performance, in accordance with Hypothesis 3. A possible explanation of this finding could be that when players strive and work for the team, positive outcomes, such as better performance, are achieved. Similar associations were previously pointed out by Boyd et al. (2014), suggesting that the collective effort could improve group performance. Boyd et al. stated that sacrifice could improve performance because each player fulfills an important and special role for the team, players are attracted to the team to achieve collective goals, accepting mistakes as a normal learning process, and focusing on generating player cohesiveness on and off the field. Therefore, it seems logical to conclude that athletes who perceive their teams' optimal inside sacrifice, where all players work for the team, also perceive better results in competitions.

Fourthly, the present study also went beyond the direct association between athlete leadership and possible positive benefits and attempted to explain the underlying indirect mechanisms that help to improve leaders' positive impact on the team's functioning. When analyzing inside sacrifice as a mediator between perceived athlete leadership quality and perceived performance (Hypothesis 4), the results showed that, when players perceive high-quality leaders in the team and strive to meet the challenges of competition, they report achieving higher performance. Prior literature indicated that the greater the confidence of players in their team's abilities, the more effort they exert, and the better they perform (Silver and Bufanio, 1996; Greenlees et al., 1999; Stajkovic et al., 2009; Cronin et al., 2015). This finding is also in line with previous research showing that the positive relationship between several group processes was stronger when there is a greater internal sacrifice by the players (e.g., transformational leadership behaviors and task cohesion; Cronin et al., 2015). Therefore, the players also perceive that athlete leadership quality enhances their performance, especially when they perceive that everyone is making a great sacrifice. Athlete leadership can be fulfilled by several players, making all the players feel closer to these leaders, driven by them, and more willing to sacrifice themselves for the team. This process of support, encouragement, and sacrifice are undoubtedly elements that promote better perceived performance. These findings are relevant because researchers have not yet examined the mediating function of inside sacrifice between perceived athlete leadership quality and perceived performance. Definitely, perceptions of high inside sacrifice seem relevant to improve the relationship between perceived athlete leadership quality and performance perceptions.

Finally, concerning Hypothesis 5, findings showed that team identification acted as a mediator in the relationship between perceived athlete leadership quality and inside sacrifice (H5a), and, together with inside sacrifice, in the relationship of perceived athlete leadership quality and perceived performance (H5b). In other words, perceived athlete quality leadership is associated with team identification (“we,” “us”), which produces stronger inside sacrifice and better perceived performance. Previous studies established that team identification also acted as a mediator in the relationship between perceived athlete leadership quality and other outcomes (i.e., collective efficacy, group cohesion, etc.; Fransen et al., 2014, 2015a, 2016b), suggesting that leaders can influence team functioning especially when team members feel identified with their team. In our study, we observed that when players identified with their team, they were more likely to sacrifice themselves for their team. When athletes play on a team with which they do not feel identified, in moments of weakness, their sacrifice may decrease. In this regard, the present research advances previous studies, analyzing the mediator function of team identification in other variables.

Also, as athlete leaders' work for the team (i.e., they create a shared sense of “we” and “us” within the group; Haslam et al., 2011; Steffens et al., 2014) strengthens team members' identification with the team (Haslam et al., 2011) and facilitates shared success (Fransen et al., 2014), perceived athlete leadership quality may have increased team identification and motivation to exert more effort for the team, thereby, ultimately enhancing their perceived performance (Haslam et al., 2000). This result implies the existence of other mechanisms through which perceived athlete leadership quality can positively affect players' performance perceptions. Hence, team identification, in conjunction with players' inside sacrifice, may be essential to improve perceived performance.

## Limitations and Future Research Directions

This research is the first study of the underlying mechanisms that explain the relationship between perceived athlete leadership quality, team identification, inside sacrifice, and perceived performance. We aimed to provide initial evidence for future investigations. However, some limitations should be commented on when interpreting the findings of an investigation of this kind, which may be important to improve future studies.

First, as our findings were correlational and we used a cross-sectional design, we cannot make causal inferences between the constructs included in this research. Future investigations could address the relationship between variables considered in the current study through experimental or quasi-experimental designs, for instance, including several measures across a competitive season to test fluctuations in the variables related to athlete leadership quality. Second, another limitation is the measurement of perceived performance. Although the instrument used in the present study to assess performance has been previously used with positive evidence, it only had two items. Therefore, due to performance is a multifactorial variable, future research should use more detailed scales or an instrument that jointly contemplates objective and perceived performance. Third, another issue of our work is the small sample size in basketball or volleyball. More research is needed with a larger number of players in these sports and others. Besides, due to the small number of female players, we did not consider gender differences. Therefore, for future studies, we recommend determining gender differences in the associations between the variables under investigation. Finally, although previous studies have analyzed the athlete leadership quality reported by players, we recommend examining the leadership quality using a qualitative methodology (e.g., observational design) to analyze the particular mechanisms and behaviors in these leaders.

## Practical Implications

Several recommendations or practical applications can be drawn as strategies to apply in real competitive contexts. The findings suggest that coaches and sports psychologists should carefully consider the perceptions of leaders' quality to achieve teams' better inside sacrifice and performance perceptions. Coaches should identify athlete leaders within the team to help develop their leadership skills. For example, coaches should stimulate their athlete leaders through individual interviews so they will express positive behaviors in training sessions and matches and show their enthusiasm for the team, striving in each competitive situation. As a result, coaches should be aware that, if they take care of leaders' quality and strengthen this type of leadership, they will achieve better team functioning. In particular, the mediating role of team identification shows the need for coaches to develop their players' feelings of being a part of the group, promoting the use of the term “us” and the achievement of collective objectives. Our model also highlights the important role of inside sacrifice, and the need to reward players' efforts to improve their performance in competition. Coaches could help players to know which roles and sacrifices they expect from them and teach them how to increase these behaviors in practice sessions and competitions. In short, this work could serve as a support for professionals working in these sports, showing the importance of perceived athlete leadership quality and promoting a shared leadership structure that is not yet observed in many team sports.

## Conclusions

This research reveals the benefits of perceived athlete leadership quality, represented by inside sacrifice and perceived performance. First, it has been reported that high-quality athlete leaders are positively associated with inside sacrifice and performance. Second, teams with higher inside sacrifice are more likely to achieve better team performance. Third, it was shown that inside sacrifice is a mediator of the association between perceived athlete leadership quality and performance perceptions. We also conclude that team identification plays an essential mediation role in all these relationships (i.e., athlete leadership with inside sacrifice and athlete leadership with perceived performance). Thus, this research advances the study of athlete leadership quality, including relevant findings of different positive outcomes that can optimize team functioning.

## Data Availability Statement

The raw data supporting the conclusions of this article will be made available by the authors, without undue reservation.

## Ethics Statement

The studies involving human participants were reviewed and approved by 239/2019. Written informed consent to participate in this study was provided by the participants' legal guardian/next of kin.

## Author Contributions

ML-G was responsible for conducting analysis and writing the first draft. MT-S was responsible for the data collection. FL and IR-B both set up the design of the study and consistently provided feedback on the content, layout, and writing style. IR-B participated in idea development. All authors contributed to the article and approved the submitted version.

## Conflict of Interest

The authors declare that the research was conducted in the absence of any commercial or financial relationships that could be construed as a potential conflict of interest.
