# Supplementary material for: Is Perceived Athlete Leadership Quality Related to Inside Sacrifice and Perceived Performance in Team Sports? The Mediating Role of Team Identification
Source: Front Psychol. 2021 Jun 21;12:662250. doi: 10.3389/fpsyg.2021.662250 (PMC8255367; doi:10.3389/fpsyg.2021.662250)
Supplement: Supplementary file 1 [file Table_1.docx]

**Supplemental Table 1**. *Means, Standard Deviations and Bivariate Correlations of the Subscales Corresponding to the Variables under Investigation.*

|  | *M* | *SD* | 1 | 2 | 3 | 4 | 5 | 6 | 7 | 8 |
| --- | --- | --- | --- | --- | --- | --- | --- | --- | --- | --- |
| 1. Athlete’ task leadership quality | 6.07 | .91 | - |  |  |  |  |  |  |  |
| 2. Athlete’ social leadership quality | 6.01 | 1.14 | .54^***^ | - |  |  |  |  |  |  |
| 3. Athlete’ motivational leadership quality | 6.21 | .95 | .61^***^ | .62^***^ | - |  |  |  |  |  |
| 4. Athlete’ external leadership quality | 6.03 | 1.19 | .46^***^ | .66^***^ | .52^***^ | - |  |  |  |  |
| 5. Team identification | 4.78 | .46 | .27^***^ | .21^***^ | .28^***^ | .13^*^ | - |  |  |  |
| 6. Personal sacrifice | 8.05 | .94 | .20^***^ | .21^***^ | .28^***^ | .25^***^ | .32^***^ | - |  |  |
| 7. Teammate sacrifice | 7.46 | 1.29 | .27^***^ | .26^***^ | .30^***^ | .25^***^ | .40^***^ | .66^***^ | - |  |
| 8. Individual performance | 3.97 | .88 | .19^**^ | .17^*^ | .21^***^ | .20^***^ | .34^***^ | .24^***^ | .30^***^ | - |
| 9. Team performance | 4.28 | .72 | .36^***^ | .30^***^ | .32^***^ | .34^***^ | .26^***^ | .26^***^ | .40^***^ | .39^***^ |

*Note*. ^*^*p* < .05, ^**^*p* < .01, ^***^*p* < .001.
